# Supplementary material for: HIVprotI: an integrated web based platform for prediction and design of HIV proteins inhibitors
Source: J Cheminform. 2018 Mar 9;10:12. doi: 10.1186/s13321-018-0266-y (PMC5845081; doi:10.1186/s13321-018-0266-y)
Supplement: Supplementary file 2 — Additional file 2. Source code of HIVProtI web server. [file 13321_2018_266_MOESM2_ESM.zip › HIVprotI_Source-code/hivprotI/weka-3-6-6/doc/weka/attributeSelection/package-tree.html]

weka.attributeSelection Class Hierarchy


---


|  |  |  |
| --- | --- | --- |
| **Overview** \| **Package** \| Class \| **Tree** \| **Deprecated** \| **Index** \| **Help** \| **Weka's home** | |  |
| **PREV**   **NEXT** | **FRAMES**    **NO FRAMES**     **All Classes** |  |


---


## Hierarchy For Package weka.attributeSelection

**Package Hierarchies:**: All Packages

---

## Class Hierarchy

- java.lang.Object
  - weka.attributeSelection.**ASEvaluation** (implements weka.core.CapabilitiesHandler, weka.core.RevisionHandler, java.io.Serializable)
    - weka.attributeSelection.**AttributeSetEvaluator**- weka.attributeSelection.**CfsSubsetEval** (implements weka.core.OptionHandler, weka.attributeSelection.SubsetEvaluator, weka.core.TechnicalInformationHandler)- weka.attributeSelection.**ChiSquaredAttributeEval** (implements weka.attributeSelection.AttributeEvaluator, weka.core.OptionHandler)- weka.attributeSelection.**ConsistencySubsetEval** (implements weka.attributeSelection.SubsetEvaluator, weka.core.TechnicalInformationHandler)- weka.attributeSelection.**CostSensitiveASEvaluation** (implements weka.core.OptionHandler, java.io.Serializable)
              - weka.attributeSelection.**CostSensitiveAttributeEval** (implements weka.attributeSelection.AttributeEvaluator, weka.core.OptionHandler, java.io.Serializable)- weka.attributeSelection.**CostSensitiveSubsetEval** (implements weka.core.OptionHandler, java.io.Serializable, weka.attributeSelection.SubsetEvaluator)
              - weka.attributeSelection.**FilteredAttributeEval** (implements weka.attributeSelection.AttributeEvaluator, weka.core.OptionHandler, java.io.Serializable)- weka.attributeSelection.**FilteredSubsetEval** (implements weka.core.OptionHandler, java.io.Serializable, weka.attributeSelection.SubsetEvaluator)- weka.attributeSelection.**GainRatioAttributeEval** (implements weka.attributeSelection.AttributeEvaluator, weka.core.OptionHandler)- weka.attributeSelection.**HoldOutSubsetEvaluator** (implements weka.attributeSelection.SubsetEvaluator)
                      - weka.attributeSelection.**ClassifierSubsetEval** (implements weka.attributeSelection.ErrorBasedMeritEvaluator, weka.core.OptionHandler)
                      - weka.attributeSelection.**InfoGainAttributeEval** (implements weka.attributeSelection.AttributeEvaluator, weka.core.OptionHandler)- weka.attributeSelection.**OneRAttributeEval** (implements weka.attributeSelection.AttributeEvaluator, weka.core.OptionHandler)- weka.attributeSelection.**ReliefFAttributeEval** (implements weka.attributeSelection.AttributeEvaluator, weka.core.OptionHandler, weka.core.TechnicalInformationHandler)- weka.attributeSelection.**SVMAttributeEval** (implements weka.attributeSelection.AttributeEvaluator, weka.core.OptionHandler, weka.core.TechnicalInformationHandler)- weka.attributeSelection.**SymmetricalUncertAttributeEval** (implements weka.attributeSelection.AttributeEvaluator, weka.core.OptionHandler)- weka.attributeSelection.**UnsupervisedAttributeEvaluator** (implements weka.attributeSelection.AttributeEvaluator)
                                  - weka.attributeSelection.**LatentSemanticAnalysis** (implements weka.attributeSelection.AttributeTransformer, weka.core.OptionHandler)- weka.attributeSelection.**PrincipalComponents** (implements weka.attributeSelection.AttributeTransformer, weka.core.OptionHandler)
                                  - weka.attributeSelection.**UnsupervisedSubsetEvaluator** (implements weka.attributeSelection.SubsetEvaluator)- weka.attributeSelection.**WrapperSubsetEval** (implements weka.core.OptionHandler, weka.attributeSelection.SubsetEvaluator, weka.core.TechnicalInformationHandler)
    - weka.attributeSelection.**ASSearch** (implements weka.core.RevisionHandler, java.io.Serializable)
      - weka.attributeSelection.**BestFirst** (implements weka.core.OptionHandler, weka.attributeSelection.StartSetHandler)- weka.attributeSelection.**ExhaustiveSearch** (implements weka.core.OptionHandler)- weka.attributeSelection.**GeneticSearch** (implements weka.core.OptionHandler, weka.attributeSelection.StartSetHandler, weka.core.TechnicalInformationHandler)- weka.attributeSelection.**GreedyStepwise** (implements weka.core.OptionHandler, weka.attributeSelection.RankedOutputSearch, weka.attributeSelection.StartSetHandler)- weka.attributeSelection.**LinearForwardSelection** (implements weka.core.OptionHandler, weka.attributeSelection.StartSetHandler, weka.core.TechnicalInformationHandler)- weka.attributeSelection.**RaceSearch** (implements weka.core.OptionHandler, weka.attributeSelection.RankedOutputSearch, weka.core.TechnicalInformationHandler)- weka.attributeSelection.**RandomSearch** (implements weka.core.OptionHandler, weka.attributeSelection.StartSetHandler, weka.core.TechnicalInformationHandler)- weka.attributeSelection.**Ranker** (implements weka.core.OptionHandler, weka.attributeSelection.RankedOutputSearch, weka.attributeSelection.StartSetHandler)- weka.attributeSelection.**RankSearch** (implements weka.core.OptionHandler, weka.core.TechnicalInformationHandler)- weka.attributeSelection.**ScatterSearchV1** (implements weka.core.OptionHandler, weka.core.TechnicalInformationHandler)- weka.attributeSelection.**SubsetSizeForwardSelection** (implements weka.core.OptionHandler)
      - weka.attributeSelection.**AttributeSelection** (implements weka.core.RevisionHandler, java.io.Serializable)- weka.attributeSelection.**BestFirst.Link2** (implements weka.core.RevisionHandler, java.io.Serializable)- weka.core.**Check** (implements weka.core.OptionHandler, weka.core.RevisionHandler)
            - weka.core.**CheckScheme**
              - weka.attributeSelection.**CheckAttributeSelection**
            - weka.attributeSelection.**ConsistencySubsetEval.hashKey** (implements weka.core.RevisionHandler, java.io.Serializable)- weka.core.**FastVector** (implements weka.core.Copyable, weka.core.RevisionHandler, java.io.Serializable)
                - weka.attributeSelection.**BestFirst.LinkedList2**- weka.attributeSelection.**LFSMethods.LinkedList2**
                - weka.attributeSelection.**LFSMethods** (implements weka.core.RevisionHandler)- weka.attributeSelection.**LFSMethods.Link2** (implements weka.core.RevisionHandler, java.io.Serializable)- weka.attributeSelection.**ScatterSearchV1.Subset** (implements java.io.Serializable)

## Interface Hierarchy

- weka.attributeSelection.**AttributeEvaluator**- weka.attributeSelection.**AttributeTransformer**- weka.attributeSelection.**ErrorBasedMeritEvaluator**- weka.attributeSelection.**RankedOutputSearch**- weka.attributeSelection.**StartSetHandler**- weka.attributeSelection.**SubsetEvaluator**

---


|  |  |  |
| --- | --- | --- |
| **Overview** \| **Package** \| Class \| **Tree** \| **Deprecated** \| **Index** \| **Help** \| **Weka's home** | |  |
| **PREV**   **NEXT** | **FRAMES**    **NO FRAMES**     **All Classes** |  |


---
